# Diagnostic Accuracy of Artificial Intelligence for Detection of Intracranial Haemorrhage on Non-contrast CT Head: A Systematic Review

**DOI:** 10.7759/cureus.111617

**Published:** 2026-06-27

**Authors:** Heramba Vigneshwar Sachithananthan, Anusha Chitravanshi, Ashwin Umasankar, Indrajit Banerjee

**Affiliations:** 1 Urology, Oxford University Hospitals NHS Foundation Trust, Oxford, GBR; 2 Medicine, Sir Seewoosagur Ramgoolam Medical College, Belle Rive, MUS; 3 Medicine, Government Medical College and Hospital, Thiruvallur, Thiruvallur, IND; 4 Pharmacology, Sir Seewoosagur Ramgoolam Medical College, Belle Rive, MUS

**Keywords:** artificial intellegence in health care, artificial intelligence (ai), convolutional neural network, ct (computed tomography) imaging, ct scan head, deep learning artificial intelligence, diagnostic accuracy of ai, intracranial hemorrhage (ich), machine learning, neural networks (nns)

## Abstract

Intracranial haemorrhage (ICH) is a life-threatening condition requiring rapid diagnosis to improve clinical outcomes. Non-contrast computed tomography (CT) is the primary imaging modality; however, increasing workload and diagnostic variability may lead to delays. Artificial intelligence (AI) has emerged as a potential tool to enhance detection. This systematic review evaluates the diagnostic accuracy of AI algorithms for detecting ICH on non-contrast CT.

This systematic review was conducted in accordance with Preferred Reporting Items for Systematic Reviews and Meta-Analyses (PRISMA 2020) guidelines, and the research protocol was registered in PROSPERO (CRD420261320521). A comprehensive search of PubMed, Cochrane Library, CENTRAL, ScienceDirect, and Google Scholar was performed. Diagnostic accuracy studies assessing AI-based detection of ICH on CT were included. Data extraction and study selection were conducted independently. Validated tools, including Quality Assessment of Diagnostic Accuracy Studies-2 (QUADAS-2) and Grading of Recommendations Assessment, Development and Evaluation (GRADE), were additionally utilised to assess risk of bias and certainty of evidence.

A total of 25,528 records were identified through database searching (PubMed: 392; Google Scholar: 17,900; ScienceDirect: 7,218; Cochrane Library: 18). A total of eight studies, which met the inclusion criteria, were included, demonstrating variability in AI models, datasets, and validation methods. Overall, AI algorithms showed high diagnostic performance, with sensitivities ranging from 0.73 to 0.95 and specificities from 0.80 to 0.98. Studies using larger datasets reported higher accuracy. However, heterogeneity in study design and reference standards was significant.

AI demonstrates promising diagnostic accuracy for ICH detection, particularly as a triage tool. However, variability and low-quality evidence limit generalizability. AI should be used alongside radiologists, with further prospective studies required before widespread clinical implementation.

## Introduction and background

Intracranial haemorrhage (ICH) is a life-threatening condition associated with serious morbidity and mortality, causing around two million strokes globally each year [1], out of which 60% of the patients present with increased blood pressure [2]. Nearly half of the deaths occur within the first 24 hours of injury, portraying the need to understand the pathophysiology responsible for it [3]. Despite this, the doctors primarily depend on routine non-contrast computed tomography (NCCT) due to unclear and atypical symptoms of the patient, causing delays in proper management [1].

In clinical settings, both stat and routine imaging assessments receive a large number of ICH cases from multiple departments [4]. CT is vital for trauma as well as non-trauma patients, making it a key imaging technique for severe central nervous system-related conditions like ICH, cranial fractures, cerebral microhaemorrhage and raised intracranial pressure [5-7]. Apart from this, satisfactory results were obtained in detecting diabetic retinopathy, pulmonary nodules, and skin cancer [4]. While MRI is used alongside as a secondary diagnostic method, radiologists greatly rely on CT due to its advancements like definitive diagnosis and improved image resolution, making it the primary imaging modality [5,7]. 

There are various underlying pathologies responsible for the development of ICH, mainly involving vasculitis, cerebral amyloid angiopathy, and dural arteriovenous fistula. Hypertension, trauma and hemorrhagic conversion of already existing infarction are also risk factors [6]. ICH can either be intra-axial (within the brain parenchyma) or extra-axial (outside the brain parenchyma) [4], with the following subtypes: intraventricular, intraparenchymal (intra-axial) subdural, subarachnoid extradural (extra-axial) and cranial vault fractures, each presenting a typical imaging finding. Additionally, the complexities of the anatomy of the skull make the pathologies of this region prone to neglect [8]. Subdural haemorrhage was found to be the most challenging to detect due to its erratic shape, size, and location. It also shares similar features like extradural haemorrhage and might show imaging intensity values identical to surrounding structures, contributing to its poor detection and misclassification [6]. It has been noticed that intraventricular haemorrhage shows the best result while extradural haemorrhage has the least detection [6,9]. Small ICHs have a good prognosis and limited intracranial expansion with proper medical attention [6,10], putting forward the need for accurate diagnosis followed by management which can be achieved by automatic identification of these subtypes using annotated imaging datasets like the RSNA 2019 (Radiological Society of North America, Oak Brook, IL, USA) head CT dataset [11] and Improved YOLOv8 (Ultralytics Inc., Austin, TX, USA) network, that overcomes some limitations of the former, like inability to localize bleeding and only classifying it [9]. 

Latest innovations focus on automatic detection of ICH and aim to reduce workload on radiologists by serving as a tool for re-evaluation, rapid detection, and minimizing the chances of error. For example, a deep learning artificial intelligence (AI) tool detected a case of ICH accurately within 39 minutes in an 88-year-old female patient with mental status changes for one week, which was previously regarded as an adverse effect of alprazolam [4]. Platforms such as convolutional neural networks (CNNs) [5,6,9], Aidoc (Aidoc Medical Ltd., Tel Aviv, Israel), and AutoStroke (Canon Medical Systems Corporation, Otawara, Japan) have been successfully evaluated to be used as a diagnostic tool [1,11]. It is essential to validate and verify AI tools through different clinical scenarios before integration into clinical practice for intracranial haemorrhage detection [12,13].

Despite the improvements, AI algorithms come with limitations related to diagnostic performance in different clinical scenarios, image quality, low sensitivity and low positive predictive value [14]. The workload still remains intense on the medical staff, leading to missed lesions and diagnostic errors [6,7,15]. Many AI-assisted systems have been experimented for diagnosis of ICH [16,17] and can be used to overcome the challenges and serve as an important diagnostic tool to support clinicians [9]. However, since there are increased reports of false negatives, AI-based tools can serve as a medium for secondary review instead of full generalization [5]. Despite the growing number of studies evaluating AI algorithms for the detection of ICH on NCCT, the reported diagnostic performance varies considerably across different models, datasets, and clinical settings. Furthermore, there remains limited consolidated evidence comparing the accuracy, sensitivity, specificity, and clinical applicability of these AI systems in routine radiological practice. A reliable AI tool can enhance the work of radiologists during the routine diagnosis (which takes longer as compared to stat) and triage to detect the complexities in less time with fewer errors, making it suitable to be used as a second review. It can also reduce the increasing workload on junior doctors, especially in the absence of a specialist during the night shift, which contributes to delayed reporting and oversight of ICH [16]. Therefore, a systematic review is necessary to critically evaluate and synthesise the currently available evidence regarding the diagnostic performance and potential clinical utility of AI in ICH detection. The primary objective of this systematic review is to evaluate the diagnostic accuracy and clinical applicability of AI algorithms in detecting ICH on NCCT imaging by analysing the currently available published literature.

## Review

Methodology

Study Design

Preferred Reporting Items for Systematic Reviews and Meta-Analyses (PRISMA) 2020 guidelines were used to conduct this systematic review [18]. The review protocol was registered in the International Prospective Register of Systematic Reviews (PROSPERO) prospectively under registration number CRD420261320521.

Inclusion Criteria

Only prospective and retrospective studies were included. Studies gauging the performance of AI algorithms for the detection of Intracranial haemorrhage on NCCT head imaging as well as metrics such as sensitivity, specificity, and/or area under the receiver operator characteristic curve (AUC) were considered. Commercial, non-commercial, and studies using institutional and/or public datasets were eligible. Additionally, studies were mandated to correlate AI algorithm results against an accepted reference standard such as radiologist interpretation, consensus radiology reporting, clinical follow-up confirmation, surgical findings, or multidisciplinary diagnosis.

Exclusion Criteria

Review articles, meta-analyses, case reports, editorials, commentaries, conference abstracts lacking sufficient diagnostic data, and Animal studies were not included. Studies not evaluating NCCT or without a diagnostic performance outcome/diagnostic validation were not considered eligible for inclusion.

Information Sources 

An extensive search was conducted on PubMed, Cochrane, ScienceDirect and Google Scholar databases from 26 February 2026 to 10 March 2026.

Search Strategy

The search strategy included combinations of keywords and medical subject headings (MeSH) terms such as “artificial intelligence”, “machine learning”, “deep learning”, “neural network”, “intracranial haemorrhage”, “intracerebral haemorrhage”, “computed tomography”, and “CT Head”. Boolean operators AND/OR were used to combine search terms appropriately (Table 1).

**Table 1:** Search Strategy and Various Databases Used Summary of databases searched, keywords/MeSH terms used, search strategies, applied filters, and the number of records retrieved.

Database	Keywords / MeSH Terms Used	Search Strategy	Filters Applied	Results Retrieved	Date searched
PubMed	("Intracranial Haemorrhages"[Mesh] OR "Brain Haemorrhage"[Mesh] OR intracranial hemorrhag*[tiab] OR intracranial haemorrhag*[tiab] OR brain hemorrhag*[tiab] OR brain haemorrhag*[tiab] OR ICH[tiab] OR intracerebral hemorrhag*[tiab] OR intracerebral haemorrhag*[tiab] OR subarachnoid hemorrhag*[tiab] OR subdural hematoma[tiab] OR extradural hematoma[tiab]) AND ("Artificial Intelligence"[Mesh] OR "Machine Learning"[Mesh] OR "Deep Learning"[Mesh] OR "Neural Networks, Computer"[Mesh] OR artificial intelligence[tiab] OR machine learning[tiab] OR deep learning[tiab] OR convolutional neural network*[tiab] OR CNN[tiab] OR algorithm*[tiab] OR automated detection[tiab] OR computer-aided[tiab]) AND ("Tomography, X-Ray Computed"[Mesh] OR computed tomography[tiab] OR CT[tiab] OR CT head[tiab] OR head CT[tiab] OR brain CT[tiab] OR non-contrast CT[tiab] OR noncontrast CT[tiab] OR NCCT[tiab]) AND ("Sensitivity and Specificity"[Mesh] OR sensitivity[tiab] OR specificity[tiab] OR diagnostic accuracy[tiab] OR accuracy[tiab] OR AUC[tiab] OR area under curve[tiab] OR ROC[tiab] OR receiver operating characteristic[tiab] OR predictive value*[tiab])	Boolean search combining intracranial haemorrhage terms AND artificial intelligence terms AND computed tomography imaging terms	Humans; English language	392	26/02/2026
Cochrane Library	(intracranial hemorrhage OR intracranial haemorrhage OR ICH) AND (artificial intelligence OR machine learning OR deep learning) AND (CT OR computed tomography)	Keyword-based Boolean search combining intracranial haemorrhage terms AND artificial intelligence terms AND computed tomography terms	Humans; English language	18	08/03/2026
ScienceDirect	(intracranial hemorrhage OR intracranial haemorrhage OR ICH) AND (artificial intelligence OR machine learning OR deep learning) AND (CT OR computed tomography)	Keyword-based Boolean search combining intracranial haemorrhage terms AND artificial intelligence terms AND computed tomography terms	English language; article type filters	7,218	04/03/2026
Google Scholar	("intracranial hemorrhage" OR "intracranial haemorrhage" OR ICH) ("artificial intelligence" OR "machine learning" OR "deep learning") (CT OR "computed tomography")	Boolean keyword search combining intracranial haemorrhage terms AND artificial intelligence terms AND computed tomography terms AND diagnostic performance terms	No filters applied	17900	10/03/2026
N=25528					

Population, Intervention, Comparison, and Outcome (PICO) Framework

Table 2 summarizes the PICO framework.

**Table 2:** Population, Intervention, Comparison, and Outcome (PICO) Framework AUC, area under the curve. The framework was used to guide study selection and data extraction for this systematic review.

Component	Description
Population (P)	Patients undergoing non-contrast head CT for suspected intracranial haemorrhage
Intervention (I)	Artificial intelligence/deep learning algorithms for intracranial haemorrhage detection
Comparison (C)	Radiologist interpretation and/or standard radiology workflow
Outcome (O)	Diagnostic accuracy (sensitivity, specificity, AUC), haemorrhage subtype detection, workflow efficiency, reporting prioritization

Study Selection and Data Synthesis

All identified results were screened by title and abstract to determine eligibility. Two reviewers independently screened the results (HVS, AC). Potentially relevant studies were assessed by reading the full text articles according to predefined inclusion and exclusion criteria. Data extraction was performed by HVS and verified by AU to ensure accuracy. Any discrepancies were resolved through discussion and consensus under the guidance of the senior author (IB). All the data was subsequently filled into a data extraction sheet. Author, year of publication, study design, dataset size, reference standard and diagnostic performance metrics were included in the data extraction table. A qualitative meta-analysis was not performed due to anticipated heterogeneity across studies. 

Risk of Bias Assessment

The Quality Assessment of Diagnostic Accuracy Studies-2 (QUADAS-2) tool was used to assess the quality of the included papers. This tool evaluates risk of bias across four domains: patient selection, index test, reference standard and flow and timing [19].

Certainty of Evidence

Quality of evidence was reviewed across domains including risk of bias, consistency, directness and precision and categorised as high, moderate, low or very low certainty using the Grading of Recommendations Assessment, Development and Evaluation (GRADE) framework [20].

Results

Study Selection

**Figure 1:**
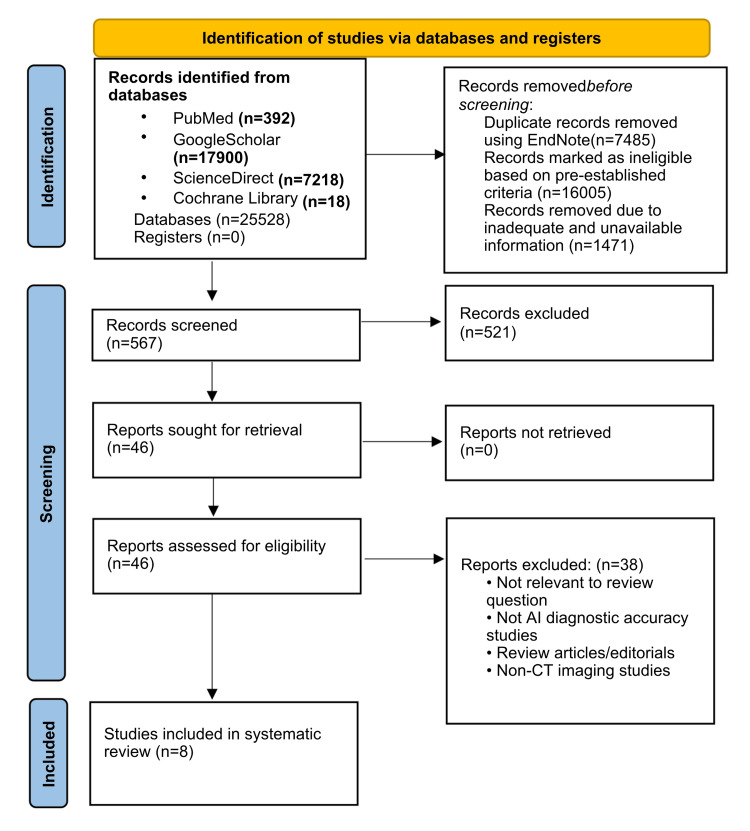
Preferred Reporting Items for Systematic Reviews and Meta-Analyses (PRISMA) 2020 Flow Diagram Flow diagram illustrating the process of study identification, screening, eligibility assessment and inclusion in the systematic review, including reasons for exclusion at each stage.

A total of 25,528 records were identified through database searching (PubMed: 392; Google Scholar: 17,900; ScienceDirect: 7,218; Cochrane Library: 18). After removal of 7,485 duplicates by using EndNote (Clarivate Plc, London, UK), 16,005 for ineligibility based on pre-established criteria and 1,471 for other reasons, 567 records remained for title and abstract screening. Of these, 521 studies were excluded due to irrelevance to the review question or inappropriate study design. A total of 46 full-text articles were assessed for eligibility, of which 38 were excluded for reasons including non-diagnostic study design, use of non-CT imaging modalities, engineering papers with no radiologist comparison and insufficient reporting of diagnostic accuracy outcomes. Ultimately, eight studies were included in the systematic review (Figure 1).

Study Characteristics

A total of eight studies published between 2018 and 2025 were included in this systematic review. The studies were conducted across multiple countries, including India, the United States, Belgium, Germany, and China. Most studies employed retrospective diagnostic accuracy or validation designs, while one study performed prospective workflow evaluation. Dataset sizes varied considerably, ranging from 302 patients to over 313,000 CT scans (Table 3).

**Table 3:** Characteristics of Included Studies Assessing Artificial Intelligence for Detection of Intracranial Haemorrhage on Non-contrast CT (NCCT) Head AP, average precision; BiFPN, bidirectional feature pyramid network; CAQ, Certificate of Added Qualification; CNN, convolutional neural network; EDH, epidural haemorrhage; FC, fully connected; ICH, intracranial haemorrhage; IPH, intraparenchymal haemorrhage; IVH, intraventricular haemorrhage; mAP, mean average precision; ML, machine learning; NPV, negative predictive value; NR, not reported; PPV, positive predictive value; RNN, recurrent neural network; RSNA, Radiological Society of North America (Oak Brook, IL, USA); SAH, subarachnoid haemorrhage; SDH, subdural haemorrhage; Wise-IoU, wise intersection over union. Proprietary and Open-Source Systems: Aidoc (Aidoc Medical Ltd., Tel Aviv, Israel); Canon AUTOStroke ML (Canon Medical Systems Corp., Otawara, Japan); ResNet18 (Microsoft Research, Redmond, WA, USA); U-Net (University of Freiburg, Freiburg, Germany); YOLOv8 (Ultralytics Inc., Austin, TX, USA).

Study	Year	Country	Study design	Center type	AI model/architecture	Dataset size	Reference standard	Haemorrhage types detected	
Chilamkurthy et al. [1]	2018	India	Retrospective diagnostic accuracy	Multicenter	Modified ResNet18 + U-Net + random forest	313,318 CT scans	Radiologist consensus	IPH, IVH, SDH, EDH, SAH	
Arbabshirani et al. [4]	2018	USA	Retrospective + clinical workflow	Multicenter	3D deep CNN ensemble (5 conv + 2 FC layers)	46,583 CT studies (~2M images)	Radiology reports	ICH (all types)	
Rava et al. [14]	2021	USA	Retrospective clinical validation	Single-center stroke center	Canon AUTOStroke ML algorithm	302 patients (200 ICH, 102 controls)	Expert clinical interpretation	IPH, IVH, SDH, SAH	
Voter et al. [15]	2021	USA	Retrospective diagnostic accuracy	Academic medical center	Aidoc FDA-cleared deep learning	3,605 NCCT scans	CAQ-certified neuroradiologist	SDH, SAH, IPH, IVH, EDH, mixed ICH	
Buls et al. [5]	2021	Belgium	Prospective workflow evaluation	Single-center	Aidoc CNN (FDA- and CE-cleared)	500 head CT scans	Consensus - 3 neuroradiologists	IPH, IVH, SDH, EDH, SAH	
Gruschwitz et al. [6]	2021	Germany	Retrospective + prospective simulation	Single-center	Dense U-Net deep learning pipeline	872 retro + 100 prospective scans	Blinded consensus - board-certified radiologists	IPH, SDH, EDH, SAH, IVH	
Wang et al. [7]	2021	China	Retrospective multicenter	Multicenter	CNN-RNN hybrid algorithm	>25,000 CT scans (RSNA + external)	Radiologist annotations	IPH, IVH, SDH, SAH, EDH	
Liu et al. [9]	2025	China	Retrospective multicenter dev + validation	Multicenter	YOLOv8 + BiFPN + Wise-IoU	8,867 train / 3,510 test (RSNA 2019+, CQ500+, PD1, PD2)	Consensus annotation - 3 radiologists (bounding-box)	IVH, IPH, SAH, SDH, EDH	

Various AI architectures were evaluated, including CNNs, Dense U-Net pipelines, CNN-recurrent neural network (RNN) hybrid models, and YOLOv8-based systems. The included studies assessed detection of multiple ICH subtypes, including intraparenchymal, intraventricular, subdural, epidural, and subarachnoid haemorrhages (Table 3). Reported sensitivities ranged from 0.73 to 0.946, while specificities ranged from 0.80 to 0.977. Several studies additionally demonstrated improvements in workflow prioritization, triage efficiency, and reporting latency through AI-assisted radiology integration (Table 4).

**Table 4:** Diagnostic Performance Outcomes of Artificial Intelligence Models for Detection of Intracranial Haemorrhage on Non-contrast CT Head. AP, average precision; AUC, area under the receiver operating characteristic curve; EDH, epidural haemorrhage; IPH, intraparenchymal haemorrhage; IVH, intraventricular haemorrhage; LR+, positive likelihood ratio; mAP, mean average precision; MCC, Matthews correlation coefficient; NPV, negative predictive value; NR, not reported; PPV, positive predictive value; SAH, subarachnoid haemorrhage; SDH, subdural haemorrhage.

Study	Year	Sensitivity / Recall	Specificity	PPV / Precision	NPV	Accuracy	AUC / performance metric
Chilamkurthy et al. [1]	2018	0.946	0.902	NR	NR	NR	0.9419
Arbabshirani et al. [4]	2018	0.73	0.8	0.64	NR	0.84	0.846
Rava et al. [14]	2021	0.93	0.93	0.85	0.98	0.94	MCC†: 0.87
Voter et al. [15]	2021	0.923	0.977	0.813	0.992	0.969 concordance	NR
Buls et al. [5]	2021	0.84	0.94	0.61	0.98	0.93	κ†: 0.65
Gruschwitz et al. [6]	2021	0.914	0.904	0.865	0.939	0.91	LR+†: 9.52
Wang et al. [7]	2021	NR	NR	NR	NR	NR	0.949-0.964
Liu et al. [9]	2025	IVH 0.787 / IPH 0.709 / SAH 0.614 / SDH 0.617 / EDH 0.454	NR	IVH 0.798 / IPH 0.827 / SAH 0.528 / SDH 0.621 / EDH 0.912	NR	NR	AP: IVH 0.852, IPH 0.820, SAH 0.574, SDH 0.639, EDH 0.558; mAP† 0.695

Risk of Bias Assessment

**Figure 2:**
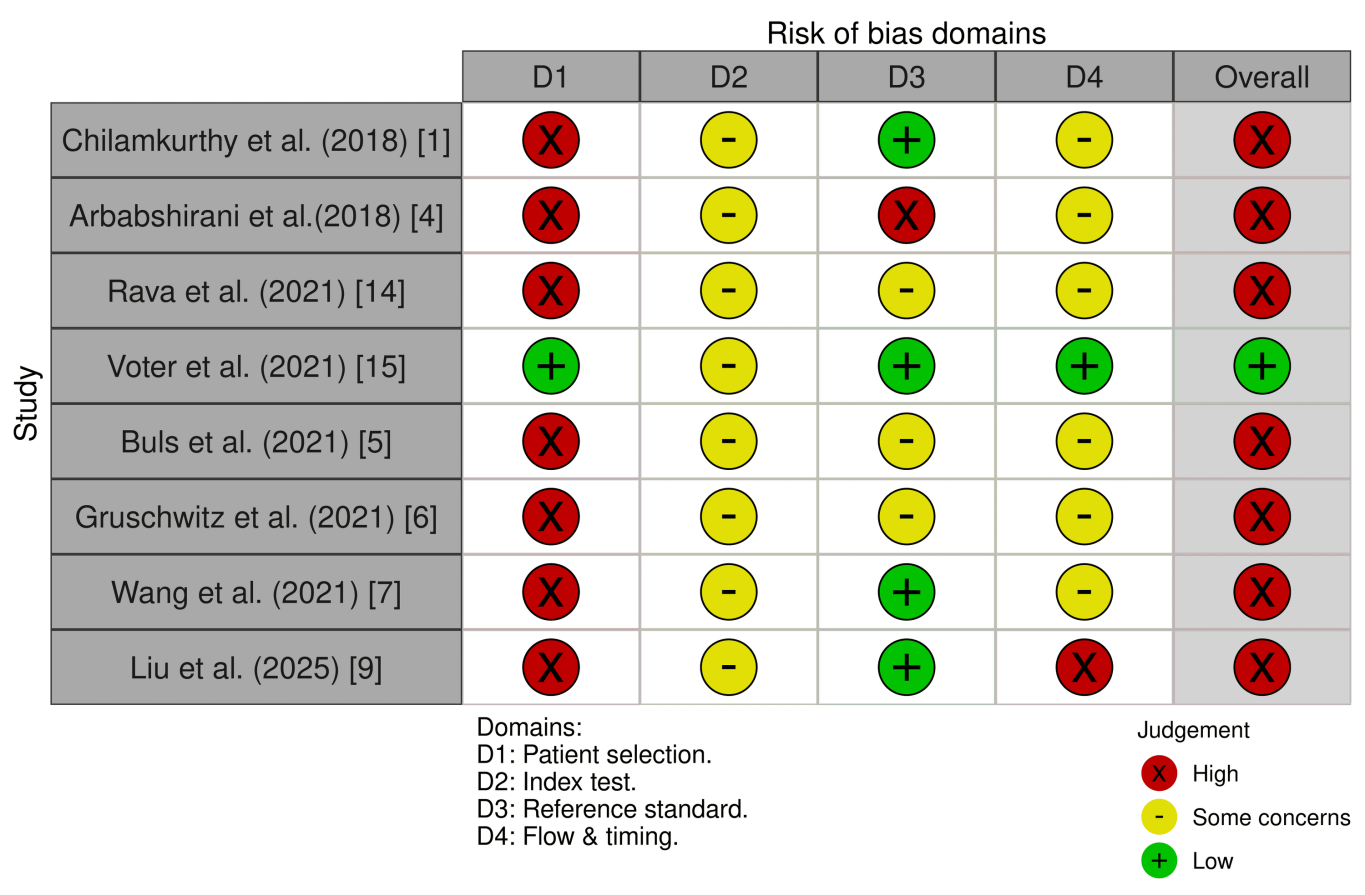
Traffic Light Plot Domains assessed were patient selection (D1), index test (D2), reference standard (D3), and flow and timing (D4).

**Figure 3:**
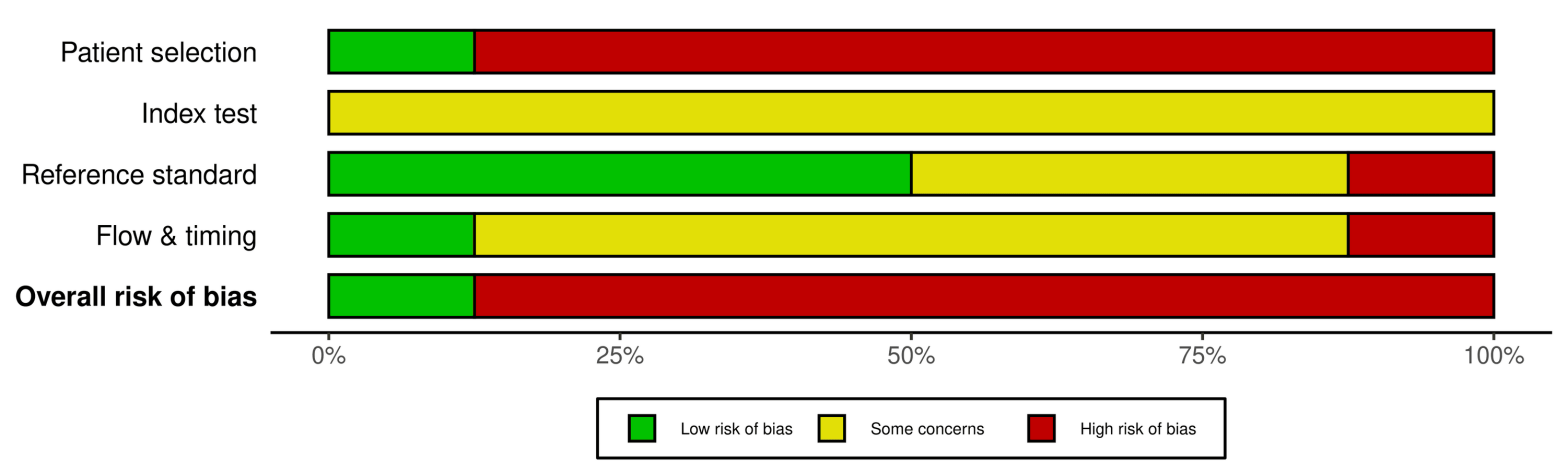
Summary Plot Green represents low risk, yellow represents some concerns, and red represents high risk of bias.

Risk of bias was assessed using the QUADAS-2 tool across four domains: patient selection, index test, reference standard, and flow and timing. Overall, the methodological quality of included studies was variable, with risk of bias most pronounced in the patient selection domain. The majority of studies were judged to be at high risk of bias in this domain due to retrospective study designs, non-consecutive sampling, and potential selection bias. The index test domain demonstrated predominantly some concerns, largely related to insufficient reporting of blinding and lack of pre-specified diagnostic thresholds. The reference standard domain showed comparatively lower risk of bias, with several studies demonstrating appropriate and clinically relevant reference standards, although some concerns remained where standardization was unclear. The flow and timing domain was generally rated as some concerns, with one study identified as high risk due to incomplete reporting of patient flow or timing between index test and reference standard. The distribution of risk of bias across studies is illustrated in Figures 2, 3. Figure 2 demonstrates a traffic light plot and Figure 3 represents summary plots. 

High risk of bias was noted in the field of patient selection in majority of the studies because of the retrospective design. The index domain showed some concerns, whereas the reference standard and flow timing presented with variable outcomes throughout the studies (Table 5).

**Table 5:** Quality Assessment of Diagnostic Accuracy Studies-2 (QUADAS-2) Risk-of-Bias Judgements Across Included Studies. Table summarizing the reasons supporting the QUADAS-2 risk-of-bias judgements for each included study.

Study	Patient Selection	Index Test	Reference Standard	Flow and Timing
Chilamkurthy et al. [1]	High - Retrospective dataset with potential selection bias	Some concerns - Insufficient detail regarding threshold specification	Low - Expert radiologist reference standard used	Some concerns - Limited reporting of patient flow
Arbabshirani et al. [4]	High - Retrospective study design	Some concerns - Index test interpretation not fully described	High - Concerns regarding reference standard methodology	Some concerns - Limited reporting of flow and timing
Rava et al. [14]	High - Retrospective patient selection	Some concerns - Limited detail regarding index test interpretation	Some concerns - Reference standard details limited	Some concerns - Incomplete reporting of patient flow
Voter et al. [15]	Low - Retrospective selection	Some concerns - Some uncertainty regarding index test reporting	Low - Appropriate reference standard used	Low - Patient flow adequately reported
Buls et al. [5]	High - Prospective selection	Some concerns - Limited description of index test conducted	Some concerns - Reference standard methodology not fully detailed	Some concerns - Insufficient flow and timing information
Gruschwitz et al. [6]	High - Retrospective workflow study	Some concerns - Limited reporting of index test interpretation	Some concerns - Reference standard details incompletely reported	Some concerns - Flow and timing details limited
Wang et al. [7]	High - Retrospective multicentre dataset	Some concerns - Threshold specification and interpretation not fully reported	Low - Expert-labelled reference standard	Some concerns - Limited reporting of patient flow and exclusions
Liu et al. [9]	High - Retrospective dataset and case selection concerns	Some concerns - Limited information regarding index test interpretation	Low - Annotated reference standard used	High - Concerns regarding flow/timing and patient exclusions

Diagnostic Accuracy Outcomes

All included studies reported diagnostic performance metrics for AI-based detection of intracranial haemorrhage on NCCT. Across studies, AI algorithms demonstrated consistently high diagnostic performance, with high sensitivity and specificity reported in most cohorts. However, there was variability in reported performance metrics, likely reflecting differences in AI model design, dataset characteristics, and validation approaches. 

Certainty of Evidence

The overall certainty of evidence, as assessed using the GRADE framework, was low. This was primarily due to concerns regarding risk of bias, particularly in patient selection, and inconsistency across studies arising from heterogeneity in AI models, datasets, and validation strategies. There were no serious concerns regarding indirectness or imprecision, as the included studies directly addressed the review question and generally included adequate sample sizes. Publication bias could not be formally assessed but remains a potential limitation.

Summary of Findings

AI algorithms demonstrate promising diagnostic accuracy for the detection of ICH on NCCT imaging. However, the overall certainty of evidence is low, and findings should be interpreted with caution given the methodological limitations and heterogeneity across included studies.

Discussion

Diagnostic Performance of AI in Intracranial Haemorrhage Detection

This systematic review demonstrates that AI algorithms exhibit promising diagnostic performance for the detection of ICH on NCCT imaging, with consistently high sensitivity reported across most included studies [1,4,14,15]. Across the included literature, AI models showed the ability to accurately detect acute intracranial bleeding while also demonstrating potential utility in workflow prioritisation and emergency radiology support. These findings are clinically significant because rapid diagnosis of intracranial haemorrhage remains essential for reducing neurological deterioration, minimising secondary brain injury, and improving overall patient outcomes [2,10]. Delayed diagnosis may contribute to increased intracranial pressure, cerebral herniation, prolonged hospitalisation, and increased mortality, particularly in emergency settings where radiologist workload is high.

Among the included studies, Chilamkurthy et al. [1] reported one of the highest sensitivities (0.946) using a modified ResNet18 CNN trained on a large multicentre dataset comprising more than 313,000 CT scans. The model was capable of detecting multiple haemorrhage subtypes simultaneously, including intraparenchymal, intraventricular, extradural, subdural, and subarachnoid haemorrhages. Similarly, Voter et al. [15] demonstrated high sensitivity (0.923) and specificity (0.977) using an FDA-cleared deep learning AI system for intracranial haemorrhage detection on emergency CT imaging. Their study additionally incorporated failure mode analysis, providing important insight into the circumstances under which AI systems may generate false positive or false negative findings. Rava et al. [14] also reported high sensitivity and specificity values (0.93 each) using the Canon AUTOStroke Solution, further supporting the diagnostic capability of AI-assisted haemorrhage detection systems.

Despite these encouraging findings, diagnostic specificity demonstrated greater variability across studies. Voter et al. [15] reported excellent specificity, Arbabshirani et al. [4] demonstrated comparatively lower specificity (0.80) in a real-world clinical workflow implementation setting. This discrepancy suggests that diagnostic performance observed in retrospective research datasets may not always directly translate into routine clinical practice. Retrospective datasets are frequently highly curated and may exclude technically challenging examinations, motion-degraded scans, or equivocal haemorrhage cases. In contrast, real-world emergency imaging environments often involve postoperative changes, chronic haemorrhage, beam-hardening artefacts, motion artefacts, variable scan quality, and complex coexisting pathology, all of which may reduce algorithmic accuracy and contribute to increased false positive rates.

Several included studies additionally demonstrated the capability of AI algorithms to classify multiple haemorrhage subtypes. Wang et al. [7] utilised a CNN-RNN hybrid algorithm and reported excellent AUC values ranging from 0.949 to 0.964 for automatic haemorrhage subtype classification. Liu et al. [9] further advanced this concept through development of an improved YOLOv8-based framework capable of haemorrhage localisation using bounding-box annotations. Their multicentre study demonstrated relatively strong performance for intraventricular and intraparenchymal haemorrhages, whereas extradural haemorrhage detection showed comparatively lower performance. Such findings are clinically relevant because haemorrhage subtype classification may directly influence neurosurgical referral, treatment planning, and prognostic assessment.

Another important observation across the included studies was the variability in diagnostic performance among different haemorrhage subtypes. Intraventricular and intraparenchymal haemorrhages generally demonstrated higher detection accuracy compared with extradural or subdural haemorrhages. This may reflect differences in attenuation characteristics, lesion morphology, haemorrhage size, and dataset prevalence. Subdural haemorrhages may be particularly challenging because they often appear as thin crescentic extra-axial collections with variable density that may resemble adjacent cortical structures or chronic collections. Extradural haemorrhages may also be difficult to detect because of their relatively lower prevalence and limited representation within training datasets.

The capability of AI to enhance the efficiency of radiologists through prioritization of diagnosis and reduction of delayed reporting has been supported by several studies. Buls et al. [5] and Gruschwitz et al. [6] supported AI integration into Triage and as a second reading tool in extensive clinical scenarios as well as to lessen the workload on radiologists

The findings of this review additionally demonstrate the rapid evolution of AI architecture within neuroradiology. Earlier investigations primarily relied on conventional convolutional neural network models, whereas more recent studies incorporated increasingly sophisticated architectures such as CNN-RNN hybrid systems, Dense U-Net pipelines, and advanced object-detection frameworks such as YOLOv8. This progression reflects ongoing efforts to improve not only detection accuracy but also lesion localisation, haemorrhage subtype classification, and real-time clinical applicability. Increasing model complexity has contributed to improved performance metrics across newer studies, although direct comparison between investigations remains challenging because of variability in datasets, validation methods, and reference standards.

The anatomical complexity of the skull base and posterior fossa represents another challenge for AI-assisted image interpretation. Bello et al. [8] highlighted the diagnostic pitfalls associated with skull base-related lesions on routine emergency head CT imaging. Dense osseous structures, beam-hardening artefacts, calcifications, and postoperative changes may complicate automated haemorrhage detection and contribute to false positive findings. Such challenges further emphasise the importance of continued radiologist oversight even when AI systems are integrated into clinical workflows.

Sources of Heterogeneity

Significant heterogeneity across the included studies represents an important challenge in interpreting the findings of this review. Variability in AI model architecture, dataset composition, haemorrhage prevalence, imaging protocols, and validation methods likely contributed to differences in reported diagnostic performance [5-7]. Studies utilising large multicentre datasets generally demonstrated higher and more stable performance metrics compared with smaller single-centre investigations. For example, Wang et al. [7] reported excellent AUC values using a large multicentre dataset, whereas smaller workflow-based studies demonstrated greater variability in specificity and predictive values.

Differences in reference standards also contributed substantially to heterogeneity. Some studies relied on consensus review by multiple neuroradiologists, while others used radiology reports or single-radiologist interpretation as the reference standard. Such methodological differences may influence sensitivity and specificity estimates and limit direct comparison between studies. Additionally, some investigations focused specifically on emergency department populations, whereas others included broader inpatient cohorts or mixed clinical populations.

Another important source of heterogeneity relates to haemorrhage subtype representation. AI systems generally demonstrated stronger performance for intraparenchymal and intraventricular haemorrhages compared with extradural and subdural haemorrhages. Variations in haemorrhage morphology, attenuation, chronicity, anatomical location, and lesion size may all influence algorithmic detection accuracy. Furthermore, image artefacts, skull base anatomy, motion degradation, and postoperative changes may introduce additional diagnostic complexity.

Clinical Implications

From a clinical perspective, the findings of this review suggest that AI has considerable potential as a triage and workflow-support tool within emergency neuroradiology. High sensitivity across multiple studies indicates that AI systems may effectively prioritise urgent CT examinations, thereby reducing reporting delays and facilitating earlier clinical intervention [1,5]. This may be particularly beneficial during overnight reporting periods, high-volume emergency department activity, or in healthcare systems experiencing radiologist shortages.

AI systems may also function as secondary readers capable of reducing perceptual oversight errors. Prospective workflow integration studies such as Buls et al. [5] demonstrated the practical feasibility of incorporating AI into routine radiology workflows. Such improvements may contribute to more rapid identification of life-threatening ICH.

However, variability in specificity and false positive rates remains clinically important. Excessive false alerts may contribute to radiologist alert fatigue, unnecessary prioritisation of non-critical examinations, and increased workload. Consequently, AI systems should currently be implemented cautiously and used as adjunctive decision-support tools rather than standalone diagnostic replacements. Human oversight remains essential to ensure patient safety and accurate clinical interpretation.

Strengths and Limitations

This systematic review has several methodological strengths. The review was conducted according to PRISMA 2020 guidelines and prospectively registered in PROSPERO, enhancing methodological transparency and reproducibility. Multiple databases were systematically searched using predefined eligibility criteria, and study selection was independently performed to minimize selection bias. Validated tools, including QUADAS-2 and GRADE, were additionally utilized to assess risk of bias and certainty of evidence.

Despite these strengths, important limitations should be acknowledged. Most included studies were retrospective in design and therefore may not fully reflect prospective real-world clinical implementation. A quantitative meta-analysis of sensitivity and specificity was not performed because included studies reported heterogeneous outcomes, evaluated different haemorrhagic subtypes, and lacked sufficient diagnostic accuracy data (2x2 contingency tables, confidence intervals and paired sensitivity specificity estimates) were unavailable. Additionally, variability in AI model architecture, dataset composition, haemorrhage prevalence, imaging protocols, and validation methods likely contributed to differences in reported diagnostic performance.

Many studies also demonstrated a high risk of bias in patient selection and reference standard domains. Furthermore, publication bias cannot be excluded because studies reporting favourable AI performance may be more likely to undergo publication.

The relatively limited number of prospective multicentre studies further reduces the strength of current evidence regarding routine clinical implementation. In addition, several included studies utilised curated datasets that may not fully represent the complexity encountered in routine emergency imaging practice.

Certainty of Evidence and Implications for Practice

Using the GRADE framework, the overall certainty of evidence was considered low to moderate because of concerns regarding heterogeneity, retrospective study design, and risk of bias. Although AI algorithms consistently demonstrated promising diagnostic performance, the current evidence base remains insufficient to support fully autonomous clinical deployment.

From a practical perspective, AI systems should currently be viewed as supportive clinical tools designed to improve workflow efficiency, prioritisation, and diagnostic assistance. Local validation within individual healthcare systems remains essential because AI performance may vary depending on scanner characteristics, imaging protocols, disease prevalence, and patient demographics. Appropriate governance frameworks, quality assurance mechanisms, and radiologist oversight are therefore necessary to ensure safe clinical integration.

Future Directions

Future research should prioritise large prospective multicentre studies evaluating AI performance within real-world clinical workflows. Standardisation of reporting frameworks, reference standards, and outcome measures would improve comparability between studies and facilitate more robust evidence synthesis. Further refinement of AI architectures may also improve performance for challenging haemorrhage subtypes such as extradural and subdural haemorrhages.

Additionally, future studies should investigate the impact of AI integration on patient-centred outcomes including reporting turnaround time, morbidity, mortality, and healthcare resource utilisation. The role of explainable AI, external validation across diverse populations, and integration with hospital information systems also warrants further investigation.

Overall, the available evidence supports the growing integration of AI-assisted haemorrhage detection into emergency neuroradiology workflows, although further prospective validation remains necessary before widespread autonomous clinical adoption can be recommended.

## Conclusions

AI shows promising diagnostic accuracy for the detection of ICH on NCCT imaging, particularly in terms of sensitivity. AI-aided tools can be useful in reducing workflow, optimizing efficiency, prioritizing cases needing immediate attention and functioning as a supplementary diagnostic support for the radiologists. However, significant heterogeneity, methodological restriction and low certainty of evidence limit the strength of current conclusions. 

To conclude, our systematic review suggests that AI tools should be used as a supportive tool within clinical workflows. Further prospective studies are required before widespread clinical implementation of AI-based systems can be recommended.
